# Exploring the dual role of B cells in solid tumors: implications for head and neck squamous cell carcinoma

**DOI:** 10.3389/fimmu.2023.1233085

**Published:** 2023-10-05

**Authors:** Jiantong Bao, Annika C. Betzler, Jochen Hess, Cornelia Brunner

**Affiliations:** ^1^ Department of Otorhinolaryngology and Head & Neck Surgery, University Medical Center Ulm, Head & Neck Cancer Center of the Comprehensive Cancer Center Ulm, Ulm, Germany; ^2^ School of Medicine, Southeast University, Nanjing, China; ^3^ Department of Otorhinolaryngology, Head and Neck Surgery, Heidelberg University Hospital, Heidelberg, Germany; ^4^ Molecular Mechanisms of Head and Neck Tumors, German Cancer Research Center (DKFZ), Heidelberg, Germany

**Keywords:** head and neck cancer, tumor-infiltrating lymphocytes, regulatory B cells, tertiary lymphoid structures, tumor microenvironment, immunotherapy

## Abstract

In the tumor milieu of head and neck squamous cell carcinoma (HNSCC), distinct B cell subpopulations are present, which exert either pro- or anti-tumor activities. Multiple factors, including hypoxia, cytokines, interactions with tumor cells, and other immune infiltrating lymphocytes (TILs), alter the equilibrium between the dual roles of B cells leading to cancerogenesis. Certain B cell subsets in the tumor microenvironment (TME) exhibit immunosuppressive function. These cells are known as regulatory B (Breg) cells. Breg cells suppress immune responses by secreting a series of immunosuppressive cytokines, including IL-10, IL-35, TGF-β, granzyme B, and adenosine or dampen effector TILs by intercellular contacts. Multiple Breg phenotypes have been discovered in human and mouse cancer models. However, when compartmentalized within a tertiary lymphoid structure (TLS), B cells predominantly play anti-tumor effects. A mature TLS contains a CD20^+^ B cell zone with several important types of B cells, including germinal-center like B cells, antibody-secreting plasma cells, and memory B cells. They kill tumor cells via antibody-dependent cytotoxicity and phagocytosis, and local complement activation effects. TLSs are also privileged sites for local T and B cell coordination and activation. Nonetheless, in some cases, TLSs may serve as a niche for hidden tumor cells and indicate a bad prognosis. Thus, TIL-B cells exhibit bidirectional immune-modulatory activity and are responsive to a variety of immunotherapies. In this review, we discuss the functional distinctions between immunosuppressive Breg cells and immunogenic effector B cells that mature within TLSs with the focus on tumors of HNSCC patients. Additionally, we review contemporary immunotherapies that aim to target TIL-B cells. For the development of innovative therapeutic approaches to complement T-cell-based immunotherapy, a full understanding of either effector B cells or Breg cells is necessary.

## 1 Introduction

Globally, head and neck squamous cell carcinoma (HNSCC) accounts for more than 870,000 new diagnoses, and 440,000 new deaths each year (1). The causes of HNSCC are either genetic alterations following environmental carcinogen exposures (i.e., smoking, alcohol), or through malignant transformation following human papillomavirus (HPV) infection (2). The majority of HPV-driven HNSCC are caused by HPV-16 infection in the oropharynx, which encompasses the base of the tongue and tonsil, whereas HNSCC driven by environmental carcinogens is more frequent in the oral cavity, hypopharynx or larynx. HNSCC patients with the anatomical site of larynx or without prior HPV infection show worse prognosis, and are more in need of multimodality treatments including both radiation and chemotherapy following surgical resection (3). Oppositely, HPV^+^ HNSCC patients have generally better prognosis compared to their HPV- counterparts, calling for treatment de-escalation for the purpose of an improved quality of life and functional outcome (4–6).

In the scenario of cancer immunity, an immunocompetent microenvironment with a high number of tumor-infiltrating lymphocytes (TILs) is often a good prognosticator. CD8^+^ cytotoxic T cells (CTLs) are primary anti-cancer cytotoxic effectors, which fight directly against tumor cells. However, the exorbitant tumor burden could also exaggerate CTL exhaustion, leading to the overexpression of a series of inhibitory receptors at the cell surface, which include programmed cell death protein 1 (PD-1), and cytotoxic T-lymphocyte associated protein 4 (CTLA-4) (7). Hence, multiple immune checkpoint blockade (ICB) reagents have been developed to reinvigorate CTL functions.

In contrast to T cells, the roles of TIL-B cells have not been fully elucidated yet. The heterogeneity, functional plasticity, and spatial variations of TIL-B cells make it even more challenging to sketch a comprehensive picture of B cell immunity in cancers. Generally, tertiary lymphoid structures (TLSs) serve as privileged sites for the aggregation of TILs and the co-activation of T and B cells. In normal physiological conditions, B cells fulfill pivotal roles in antigen processing and presentation. They can process and present antigenic peptides via both MHC class II to CD4+ T cells (8), and cross-presentation of peptide-MHC I complexes to CD8+ T cells (9–11). TIL-B cells localized within TLSs have been observed to harbor the necessary molecular machinery for effective antigen presentation to T cells (12–16).

Beyond this, TLSs house a diverse cast of effector B cell populations, including i.e. CD20^+^ germinal-center (GC) like follicular B cells, multi-valency antigen-presenting B cells, class-switched B cells, antibody-producing plasma cells, and memory B cells (17, 18). With their tremendous antibody producing capacity, plasma cells are considered as key anti-tumor effector TIL-B cells, leading the effects of complement-dependent cytotoxic, antibody-dependent cellular cytotoxicity (ADCC) and phagocytosis (ADCP). Meanwhile, the antigen-presenting B cells present antigens to effector T cells via major histocompatibility complex (MHC) molecules at the cell surface, which also aid in the anti-tumor immunity. Beside these aggregated anti-tumorigenic structures, multiple phenotypes of pro-tumorigenic B cells – collectively known as regulatory B (Breg) cells – are also found within the tumor microenvironment (TME) (19). They induce immune-suppressive cells like myeloid-derived suppressor cells (MDSCs) and regulatory T (Treg) cells, suppress effector TIL functions, and program the TME towards an immune-suppressive direction (20).

In this article, we will present a comprehensive picture of how phenotypically and functionally distinct TIL-B cells influence tumor growth and response to treatment, with a specific focus on HNSCC. We address the most important TIL-B cell populations, their functions, prognostic relevance, and related therapeutic approaches. We begin with the pro-tumorigenic Breg cells, from their phenotypes discovered yet in both murine and human cancers, the immune-suppressive mechanisms, to the relationships with clinicopathological features. Then we describe the anti-tumorigenic TIL-B cells, beginning with the fundamental concepts of TLS assembling, to the main effector TIL-B cells, their functions, and clinical relevance. We end by summarizing the current state-of-the-art of TIL-B-based immunotherapies, either by fostering TLS formation, or by eliminating the immune-suppressive functions of Breg cells.

## 2 The pro-tumor activity of Breg cells

Breg cells, a heterogenous population of B cells initially implicated in inhibiting delayed hypersensitivity reactions, are one of the primary immunosuppressive cell populations in numerous cancer types including HNSCC (21–23).

**Figure 1:**
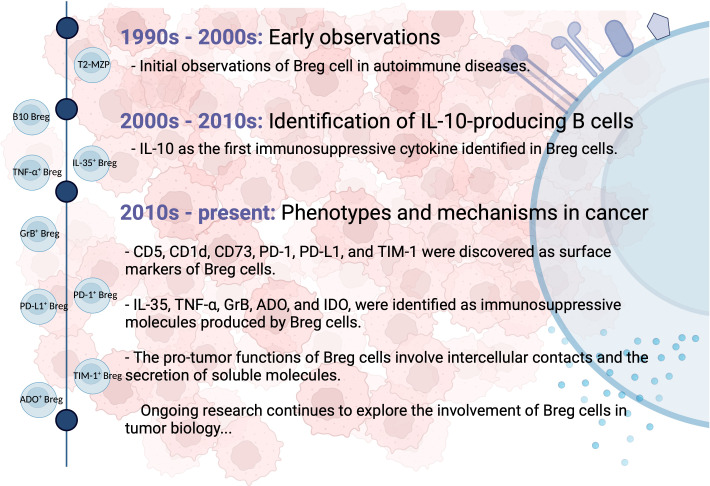
Milestones in the discovery of Breg cells. Breg cells were first identified in autoimmune diseases for their anti-inflammatory properties mediated by IL-10. They were later found in solid tumors, where they suppress anti-tumor immune responses. Multiple Breg cell phenotypes and secreted molecules have since been discovered. Ongoing studies investigate Breg cell characteristics within the tumor microenvironment.

Breg cells were initially identified in autoimmune diseases, such as collagen-induced arthritis and systemic lupus erythematosus, for their ability to reduce inflammation through the action of interleukin (IL)-10 (24). In a mouse model of experimental arthritis, transitional 2-marginal zone precursors (T2-MZP) B cells (CD21^hi^CD23^hi^CD24^hi^CD1d^hi^) were found to possess regulatory capacities and produce IL-10 (25). Additionally, IL-10-producing B cells with regulatory properties were detected within the tumor microenvironment of solid tumors such as breast and ovarian cancer, collectively referred to as B10 Bregs (26). Their presence resulted in the suppression of anti-tumor immune responses and the promotion of tumor progression by inhibiting effector T cell functions. Subsequently, other types of Breg cells with diverse surface markers and the ability to secrete different immunosuppressive molecules were discovered in multiple types of human cancers. Ongoing studies are actively investigating the phenotypic markers, secreted molecules, and mechanisms of Breg cells within the tumor microenvironment. **Figure 1** provides a schematic summary of the key milestones in the discovery of Breg cells.

Due to complex ontogeny and mode of activation, an agreement on the phenotypic and lineage trajectories of Breg cells is still lacking, as are the transcription factors that uniquely drive their development (27). The innovative strategy of employing single-cell RNA-sequencing to identify the Breg cell cluster is often unsuccessful (28, 29). Worth speculating that B cells acquire their regulatory capabilities during various stages of development and in response to certain environmental stimuli rather than Breg cells represent a distinct lineage (27, 30). Multiple local perturbations can induce Breg cells, which include hypoxia, acidosis, lipid metabolites and tumor exosomes (31–38), cytokines like IL-35, IL-21, IL-1β, IL-6 (39–42), Ca^2+^ influx (43), and activation of surface molecules including Toll-like receptors (TLRs), CD40 and B-cell receptors (BCRs) (40, 41, 44–46).

### 2.1 The immunosuppressive mechanisms of Breg cells

Breg cells promote carcinogenesis via several mechanisms. In this section, we first list the main discovered Breg phenotypes and functions of human and mouse cancer models separately in **Tables 1** and **2**. We then address the immunosuppressive mechanisms of several most important Breg cells, and also their association with the clinicopathological characteristics of HNSCC patients.

**Table 1:** Breg cell phenotypes discovered in human cancer.

Breg cell	Phenotype	Cancer	Location	Expressed molecules	Description
**B10 Breg**	CD19^+^CD24^+^CD38^+^	Invasive breast cancer	Tumor tissues, PBMCs	IL-10	PD-L1 mediated induction of Treg cells (47).
		Hepatocellular carcinoma	Tumor tissues, PBMCs	IL-10	Induce tumor proliferation and invasion via CD40/CD154 signaling pathway (48).
		Acute myeloid lymphoma	Bone marrow, PBMCs	IL-10	Increased Breg cells are predictive of a poorer prognosis (49).
		HNSCC	Tumor tissues	IL-10	B10 Breg with the CD24^hi^CD38^hi^ and CD25^hi^ phenotypes have been found in the TME. CD24^hi^CD38^hi^ B10 Breg have a higher expression of IL-10 than CD25^hi^ B10 Breg (44).
		HNSCC	Tumor-draining LNs	—	Breg cells correlate with non-metastatic LNs and low grade (50).
		Gastric cancer	Tumor tissues, PBMCs	IL-10, TGF-β	Inhibit the IFN-γ and TNF-α production of CD4^+^ T cells via IL-10; induce Treg cell proliferation via TGF-β (51).
		Multiple myeloma	Bone marrow, PBMCs	IL-10	Abrogate NK cell-mediated ADCC against multiple myeloma cells (52).
	CD19^+^CD24^hi^CD27^+^	Pancreatic cancer	PBMCs	IL-10	CD19^+^CD24^hi^CD27^+^ B10 Breg only produce IL-10, whereas CD19^+^CD24^hi^CD38^hi^ immature B cells defined in the same study produce both IL-10 and IL-35 (53).
		Esophageal squamous cell carcinoma	PBMCs	IL-10	Tumor exosomes promote B10 Breg proliferation (54).
		Gastric cancer	Tumor tissues, PBMCs	—	Inhibit the proliferation and production of IFN-γ by CD4^+^ T cells (55).
	CD19^+^CD27^+^CD10^-^	Gastric cancer	Tumor tissues, PBMCs	IL-10	Inhibit the production of IFN-γ, TNF, and IL-17 by CD4^+^ T cells; inhibit the production of IFN-γ and TNF by CD8^+^ T cells; stimulate IL-10 production by T cells (56).
	CD19^+^CD5^+^CD1d^+^	Cervical cancer, cervical intraepithelial neoplasia	PBMCs	IL-10	Inhibit the production of perforin and GrB by CD8^+^ T cells (57).
		HNSCC	Tumor-draining LNs	—	Good prognostic factor in TDLNs (50).
	CD19^+^CD5^+^	oesophageal cancer	PBMCs	IL-10	(58)
		HNSCC	Tumor-draining LNs	—	Good prognostic factor in TDLNs (50).
	CD19^+^CD20^+^	Ovarian cancer	Ascites	IL-10	Inhibit the IFN-γ production of CD8^+^ T cells via IL-10 and decreased CD80/CD86 surface expression; negatively correlate with CD4^+^FoxP3^+^ Treg cells (59).
	CD19^+^	HNSCC	Tumor tissues, LNs	IL-10	Induce resting CD4^+^ T cells to differentiate into CD4^+^FoxP3^+^ Treg cells (21).
**IL-35^+^ Breg**	CD19^+^CD24^hi^CD38^hi^	Pancreatic cancer	PBMCs	IL-10, IL-35	Cause CD8^+^ T cell malfunction via IL-35/gp130/STAT3 signaling pathway (53).
	CD20^+^	Pancreatic cancer	Tumor tissues	IL-35	Cause Treg cell expansion and CD4^+^ T cell suppression via IL-35 (60).
**GrB^+^ Breg**	CD19^+^CD38^+^CD1d^hi^IgM^+^CD147^+^	Breast, ovarian, cervical, colorectal, and prostate cancers	Tumor tissues	GrB, IL-10, IL-12	GrB^+^ Breg cells inhibit T cell proliferation and receptor degradation (57, 61).
**TIM-1^+^ Breg**	CD5^hi^CD27^-/+^CD38^+/hi^TIM-1^+^	Hepatocellular carcinoma	Tumor tissues, PBMCs	IL-10	Inhibit CD8^+^ T cell proliferation, and the production of TNF-α and IFN-γ (38).
**PD-1^+^ Breg**	CD5^hi^CD27^hi/+^CD38^dim^PD-1^+^	Hepatocellular carcinoma	Tumor tissues, PBMCs	IL-10	Cause T cell exhaustion via the PD-L1-PD-1 axis (62).
**PD-L1^+^ Breg**	CD20^+^CD27^-^PD-L1^+^	Melanoma	PBMCs	IgM, IgD	Inhibit the production of IFN-γ by T cells; express high IgM and IgD; linked to advanced tumor stages and metastasis (63).
**ADO^+^ Breg**	CD39^+^CD73^+^	HNSCC	Tumor tissues, PBMCs	ADO	Suppress the intracellular BTK and Ca^2+^ influx in effector B cells (22).
**Plasmablast**	CD19^low^CD27^hi^	Colorectal cancer	Tumor tissues	—	Gut-homing, inhibit the production of IFN-γ and TNF-α by T cells, do not promote FoxP3 expression (64).
	CD138^+^IgA^+^PD-L1^-^IL-10^+^	Prostate cancer	Tumor tissues	IL-10, TGF-β, IgA	Suppress cytotoxic CD8^+^ T cells (65).

HNSCC, head and neck squamous cell carcinoma; TME, tumor microenvironment; PBMC, peripheral blood mononuclear cells; LN, lymph node; B10 Breg, IL-10 producing regulatory B cell; T2-MZP, transitional 2-marginal zone precursor; GrB, granzyme B; ADO, adenosine; BTK, Bruton’s tyrosine kinase; ADCC, antibody-dependent cellular cytotoxicity; hi, high; dim, medium.

**Table 2:** Breg cell phenotypes discovered in mouse cancer models.

Breg type	Phenotype	Cancer	Location	Expressed molecules	Description
**B10 Breg**	CD19^+^CD21^hi^	Papilloma	Tumor tissues	IL-10	Promote TNF-α mediated squamous carcinogenesis (66).
	CD19^+^CD5^+^CD1d^hi^	Non-hodgkin lymphoma	Tumor tissues, spleen	IL-10	Inhibit lymphoma depletion and monocyte activation induced by CD20 mAbs (67).
**IL-35^+^ Breg**	CD19^+^ CD5^+^CD1d^hi^	PanIN	Tumor tissues	IL-10, IL-35	BTK signaling pathway regulated; promote tumor progression (68).
		Pancreatic cancer	Tumor tissues	IL-35	Promote tumor progression via IL-35 (69).
	CD19^+^ CD21^hi^CD5^+^CD1d^hi^	Pancreatic cancer	Tumor tissues	IL-10, IL-35	Cause CD8^+^ T cell exclusion, tumor progression, and immunotherapy resistance via the IL35/gp130/STAT3 pathway (53, 60, 70).
**PD-L1^+^ Breg**	CD19^+^PD-1^-^PD-L1^+^	4T1 breast cancer	Spleen, PBMCs	—	MDSC-induced; inhibit proliferation and production of IFN-γ by T cells (71)
**IgA^+^ Breg**	CD19^+^IgA^+^PD-L1^+^	Colorectal tumor	Tumor tissues	IL-10, TGF-β, IgA	Inhibit CD8^+^ T cell proliferation and activation (72)
	CD19^+^CD20^low^B220^low^IgA^+^PD-L1^+^	Prostate cancer	Tumor tissues	IL-10, IgA	Induce CD8^+^ T cell exhaustion, suppress cytotoxic CD8^+^ T cell activation through PD-L1 and IL-10 (65).
	CD19^+^B220^low^CD138^+^IgA^+^PD-L1^+^	Hepatocellular carcinoma	Tumor tissue	IL-10, IgA,	Suppress cytotoxic CD8^+^ T cell, cause tumor progression (73).
**T2-MZP Breg**	B220^+^CD23^+^IgM^hi^CD21^hi^	Melanoma	Tumor-draining LNs	IgM	Enriched in tumor-draining LNs; promote tumor progression (74).
**——**	CD19^+^CD81^hi^CD25^+^	4T1 breast cancer, B16F10 melanoma	Tumor tissues	TGF-β	Induce Treg cell proliferation and inhibit CD8^+^ T cell function (75).
**——**	CD86^hi^IAd^hi^CD62L^hi^LAP^+^CD44^low^PD-L1^hi^	EMT-6 breast cancer	Tumor tissues	TGF-β	Suppress T cell, Th1 cell, and NK cell proliferation; promote tumor progression (76).
**——**	Stat3^+^, CD19^+^CD25^hi^B7-H1^hi^CD81^hi^CCR6^hi^CD86^hi^CD62L^low^IgM^dim^	4T1 breast cancer	Tumor tissues	TGF-β	TGF-β mediated transformation of resting CD4^+^ T cells into FoxP3+ Treg cells; lead to tumor metastasis (77).
**——**	CD20^low^CD137^lo^	4T1 breast cancer	Tumor tissues		Cause tumor progression and metastasis (78).
**——**	CD20^+^	Prostate cancer	Tumor tissues	Lymphotoxin	Lymphotoxin-producing Breg cells can be recruited by CXCL13, which stimulate the lymphotoxin receptor on cancer cells, induce IKKα nuclear translocation and STAT3 activation, and promote cancer metastasis (79, 80).

LN, lymph node; PanIN, pancreatic intraepithelial neoplasia; PBMC, peripheral blood mononuclear cells; LN, lymph node; B10 Breg, IL-10 producing regulatory B cell; T2-MZP, transitional 2-marginal zone precursor; MDSC, myeloid-derived suppressor cell; NK cell, natural killer cell; BKT, Bruton’s tyrosine kinase; CXCL13, CXC-chemokine ligand 13; IKK, inflammation-responsive IκB kinase; hi, high; dim, medium.

#### 2.1.1 Autocrine and paracrine secretions

**Figure 2:**
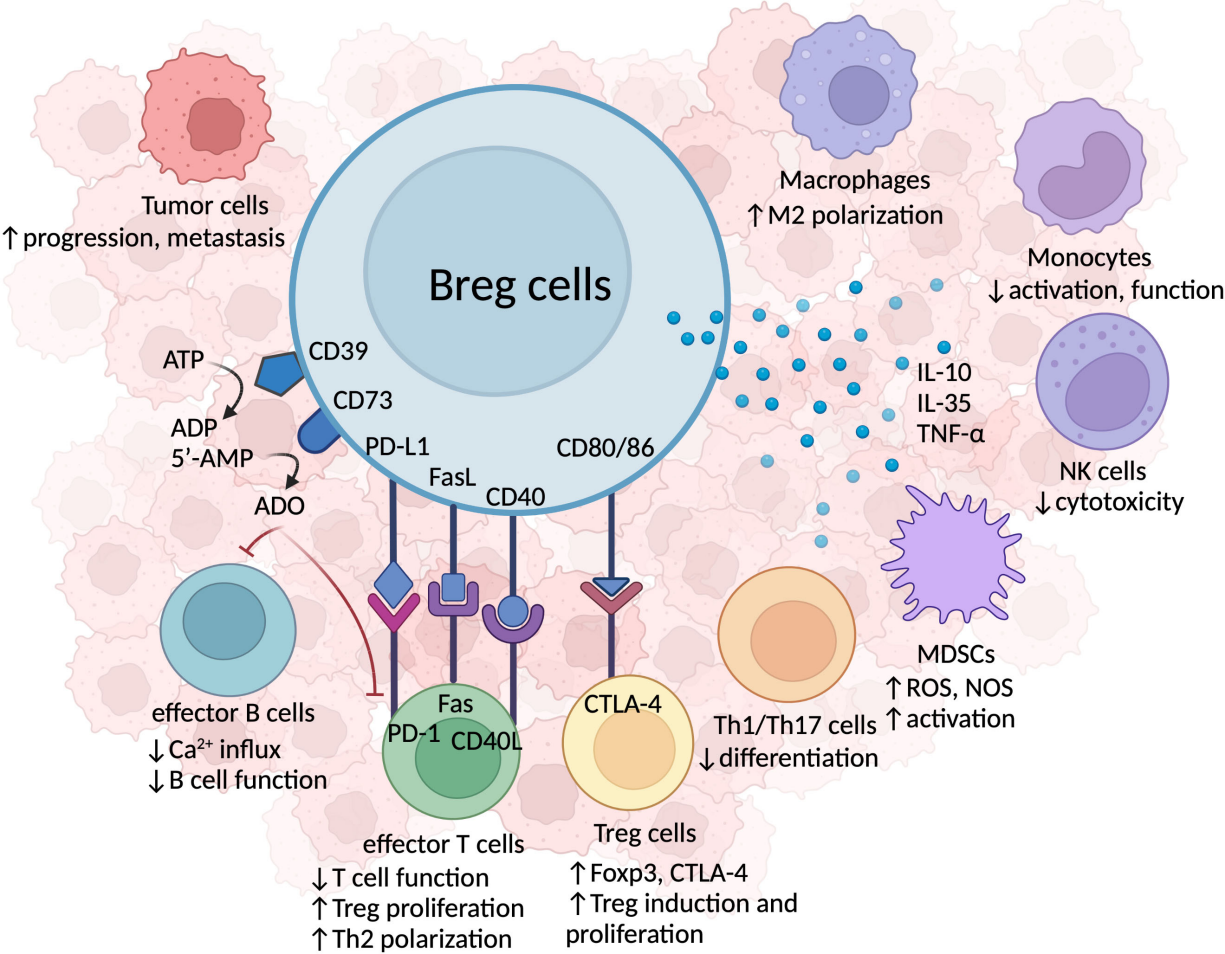
The immunosuppressive mechanisms of Breg cells in HNSCC. Breg cells secrete immunosuppressive cytokines, including IL-10, IL-35, and TNF-α, which inhibit anti-tumor immune activity, foster immunosuppressive TILs, and facilitate tumor progression and metastasis. The co-expression of CD39 and CD73 surface proteins permits Breg cells to hydrolyze ATP to adenosine (ADO), which acts on effector T and B cells, reduces Ca^2+^ influx, and results in the malfunction of effector immune cell. Breg cells also form cell-cell contacts with other TILs. Breg cells can induce anti-tumor T cell malfunction via PD-L1-PD-1 axis, or Fas/FasL binding. Breg cells and effector T cells interact via CD40/CD40L to promote Th1/Th2 cell polarization and Treg cell proliferation. Breg cells are also capable of forming cellular contacts with other immunosuppressive TILs, such as MDSCs and Treg cells. The CD80/CD86 marker expressed on Breg cells binds to CTLA-4 on Treg cells, inducing Treg cell proliferation.

Breg cells are known for their role in dampening the immune response through the secretion of endogenous anti-inflammatory molecules, such as IL-10, IL-35, and transforming growth factor-beta (TGF-β), as depicted in **Figure 2**.

In HNSCC patients, at least two kinds of IL-10-producing B10 Breg have been identified: CD19^+^CD24^hi^CD38^hi^ and CD19^+^CD25^hi^ B10 Bregs (23). Several immune-regulatory functions of B10 Bregs have been revealed: first, B10 Bregs inhibit CD4^+^ T cell differentiation into interferon (IFN)- γ and TNF-α producing helper T1 (Th1) cell and IL-17 producing helper T17 (Th17) cell (26). Second, B10 Bregs cause dendritic cells (DCs) to overexpress IL-4 and downregulate IL-12, disrupting the Th1/Th2 cell balance (81). Furthermore, B10 Bregs favorably influence the differentiation of tumor-associated macrophages (TAMs) into the M2 phenotype, and ultimately suppress effector T and natural killer (NK) cells (41, 63). B10 Bregs also dampen the activation and effector function of monocytes, and promote Treg cell development via IL-10-mediated suppressive pathways (26, 52, 67, 82–84).

Another anti-inflammatory cytokine, IL-35, promotes immunological tolerance by orchestrating the differentiation of conventional T cells to Treg cells, inducing effector T cell exhaustion, and upregulating anti-apoptotic and cell cycle genes which facilitate tumor cell growth (85, 86). Meanwhile, TGF-β converts naïve CD4^+^ T cells into Treg cells, limits effector T cell proliferation and function, and augments FoxP3 and CTLA-4 expression, hence facilitating tumor growth and metastasis (77, 87–89).

Aside from IL-10 and IL-35, unconventional Breg cells which produce other immunosuppressive molecules, including granzyme B (GrB), indoleamine 2,3-dioxygenase (IDO) and adenosine (ADO), are also detected. A unique subset of Breg cells expressing CD38, CD1d, IgM, and CD147 has been identified in various human malignancies, such as breast, ovarian, cervical, colorectal, and prostate cancers. These cells have been found to secrete regulatory molecules, including GrB, IDO, IL-12, and IL-10. Specifically, the secretion of GrB by the CD38^+^CD1d^hi^IgM^+^CD147^+^ Breg cells leads to the degradation of T-cell receptor (TCR) ζ-chain, resulting in the dampening of T cell responses (57, 61). The TIM-1-producing CD5^hi^CD24^-^CD38^+/hi^ Breg cells found in hepatocellular carcinoma patients inhibit CD8^+^ T cell proliferation, and confine the production of TNF-α and IFN-γ (38). First discovered in HNSCC patients, the ADO-producing CD39^+^CD73^+^ Breg cells suppress the activity of effector B cells by inhibiting Bruton’s tyrosine kinase (BTK) phosphorylation and Ca^2+^ influx (22). These CD39^+^CD73^+^ Breg cells also deactivate T cells in healthy volunteers via the byproducts of ATP hydrolysis, AMP and ADO, resulting in immunological escape (90).

#### 2.1.2 Intercellular interactions

Aside from producing a concoction of anti-inflammatory cytokines, Breg cells also program the immunosuppressive TME through extensive intercellular interactions with other TILs via ligand-receptor interactions including CTLA-4 — CD80/CD86, CD40 — CD40L, PD-1 — PD-L1, and Fas — FasL (**Figure 2**).

In conjunction with TGF-β, CTLA-4 — CD80/CD86 interaction enables Breg cells to form cell-to-cell contacts with Treg cells, hence boosting the expression of FoxP3 and CTLA-4 (87). The interaction between Breg cells and CD4^+^ T cells via CD40 and its ligand evokes Th1/Th2 cell polarization and Treg cell proliferation (21, 47, 51, 77, 91). Furthermore, Breg cells expressing either PD-1 or PD-L1 have been identified in cancerous tissues (62, 92). Through IL-10 signaling, PD-1^hi^ Breg cells cause CTL malfunction, and promote cancer development (62). The activation of BCL6 by TLR4 is essential for the induction of PD-1^hi^ Breg cells. Besides, PD-L1^hi^ Breg cells inhibit BCL6 overexpression in CD4^+^CXCR5^+^PD-1^+^ follicular helper T (T_FH_) cells, reduce T_FH_ proliferation, and hinder both the development of memory B cells, and the terminal differentiation of plasma cells (92). Intriguingly, multiple exhausted immune cells, including T cells, B cells, and NK cells, as well as senescence cells, have been found to have an increased expression of PD-1/PD-L1, allowing them to evade the immune surveillance of CTLs, and constantly release inflammatory cytokines (7, 93). These findings indicate that the PD-1/PD-L1-expressing Breg cells represent exhausted B cells that secrete immunosuppressive cytokines. Blocking the PD-L1-PD-1 axis, either by PD-1 blockade alone, or by concurrent blockade of IL-10 receptors and inhibitory PD-L1 receptors, have been shown to successfully reverse CTL dysfunction (93, 94).

Moreover, FasL^+^ Breg cells with the ability of promoting the death of Fas^+^ effector T cells and tumor cells have been identified (95). Since Treg cells highly express the anti-apoptotic gene c-FLIP, they are resistant to Fas-driven apoptosis. Therefore, the FasL expression by Breg cells is more likely to cause CTL elimination as opposed to Treg cells, resulting in an uneven CD8^+^ T cell/Treg cell ratio (96).

Breg cells also interact with other immunosuppressive TILs and reshape the local TME in an immunosuppressive direction. MDSCs are myeloid cells which expand under pathologic conditions, such as chronic inflammation, infection, cancer, autoimmune diseases and trauma, with potent immunosuppressive activity (97, 98). In the B-cell-deficient murine cancer model of 4T1 breast cancer and B16 melanoma, the inhibitory impact of MDSCs was greatly diminished, but adoptive transfer of tumor-evoked CD81^hi^CD25^+^CD20^low^4-1BBL^low^ Breg cells restored MDSC-mediated suppression of T cells, provoking tumor progression and metastasis (99). Coincidentally, the presence of MDSCs can confer immunosuppressive characteristics to B cells. B cells co-cultured with MDSCs for more than 24 hours were able to suppress T cell proliferation, increase IL-10 production, and decrease IFN-γ release in 4T1 breast cancer mice (71).

Last but not least, Breg cells directly promote cancer growth, invasion, and immune evasion (48, 74, 100). In the B-cell-deficient mouse model of Kras-expressing pancreatic intraepithelial neoplasia (PanIN), adoptive transfer of CD5^+^CD1d^hi^ Breg cells rescued the tumor growth deficiency (69); Meanwhile, a study using a mouse model of melanoma showed that Breg cells with B220^+^CD23^+^IgM^hi^CD21^hi^ T2-MZP phenotype specifically accumulated in tumor-draining lymph nodes (LNs) and promoted tumor growth in an IL-10-independent manner (25, 74).

### 2.2 The relation of Breg cells with clinicopathological features

While the correlation between various types of Breg cells, clinicopathological characteristics, and the prognosis of HNSCC patients remains unclear, some studies have shed light on these topics. In one study, the frequency of CD19^+^IL-10^+^ Breg cells was found to be highly associated with clinical stage, local and regional recurrence in a cohort of 46 patients with tongue squamous cell carcinoma (TSCC) (21). Moreover, higher levels of CD19^+^IL-10^+^ Breg cells and CD4^+^FoxP3^+^ Treg cells were correlated to reduced overall survival rates. Conversely, the presence of Breg cells in tumor-draining LNs appeared to be a positive prognostic indicator in HNSCC patients. In a study for non-sentinel LNs of 32 HNSCC patients, three distinct Breg phenotypes CD5^+^, CD5^+^CD1d^hi^, and CD24^hi^CD38^hi^ were analyzed by flow cytometry (50). The frequency of CD24^hi^CD38^hi^ Breg cells was significantly higher in patients with lower histological grades, while the frequency of CD5^+^ Breg cells decreased in advanced clinical stages. Considering the potential functional discrepancies involved in the tumor progression, it is possible that these Breg cell subsets perform certain immune priming functions before exerting their regulatory effects (7).

It’s also important to consider the proportion of Breg cells present within tumors. In the study on TSCC, Zhou et al. reported only 0.8% of IL-10^+^ Breg cells (21). In comparison, Lechner et al. reported a higher percentage of 2.4% IL-10^+^ Breg cells in HNSCC (23). Additionally, Hladíková et al. observed 2.7% of IL-10^+^ Breg cells in oropharyngeal cancers (101). Given that B cells are not the predominant cell type in the tumor milieu, the actual number of Breg cells is quite low. This raises the question of whether these relatively rare cells could have a significant biological impact.

Overall, amidst the diverse TILs within the TME, Breg cells discharge anti-inflammatory agents and showcase suppressive capacities to facilitate immune-regulating duties. They modulate the immune landscape of the tumor, although further research is required to thoroughly explore the relationship between Breg cells and the clinical-pathological attributes as well as the prognosis of patients with HNSCC.

## 3 Anti-tumor activity of effector B cells from TLSs

TLSs are non-encapsulated immunologically dense formations composed of lymphocytes and stromal cells, which develop in response to persistent inflammation or infection (102, 103). Their composition resembles that of secondary lymphoid organs (SLOs). Within human solid tumors, TLSs are typically found in the tumor nests and interstitium, generated as a result to persistent stimulation by tumor-associated inflammation. The aggregation of diverse TILs within a developed TLS endows it with the capacity to efficiently present tumor-associated antigens (TAAs), eliciting subsequent T and B cell effector activities.

In this section, we will initially delve into the mechanism underlying TLS formation. Subsequently, we will describe the major types of TIL-B cells located in the CD20^+^ B cell zone of TLSs, and elucidate their phenotypes, functions, and clinicopathologic significance, with a particular focus on HNSCC patients.

### 3.1 TLS formation

TLS development is a result of lymphatic neogenesis, which generates a local hub for antigen presentation and immunological activation (102). HPV^+^ HNSCC have a higher percentage of GC maturation and TLS formation within the tumor milieu (2, 104), most likely due to the presence of viral antigens throughout carcinogenesis. The episomal or integrated viral components of HPV^+^ tumors may prime for early-onset innate immune responses, and boost anti-tumor adaptive immunity (105, 106).

**Figure 3:**
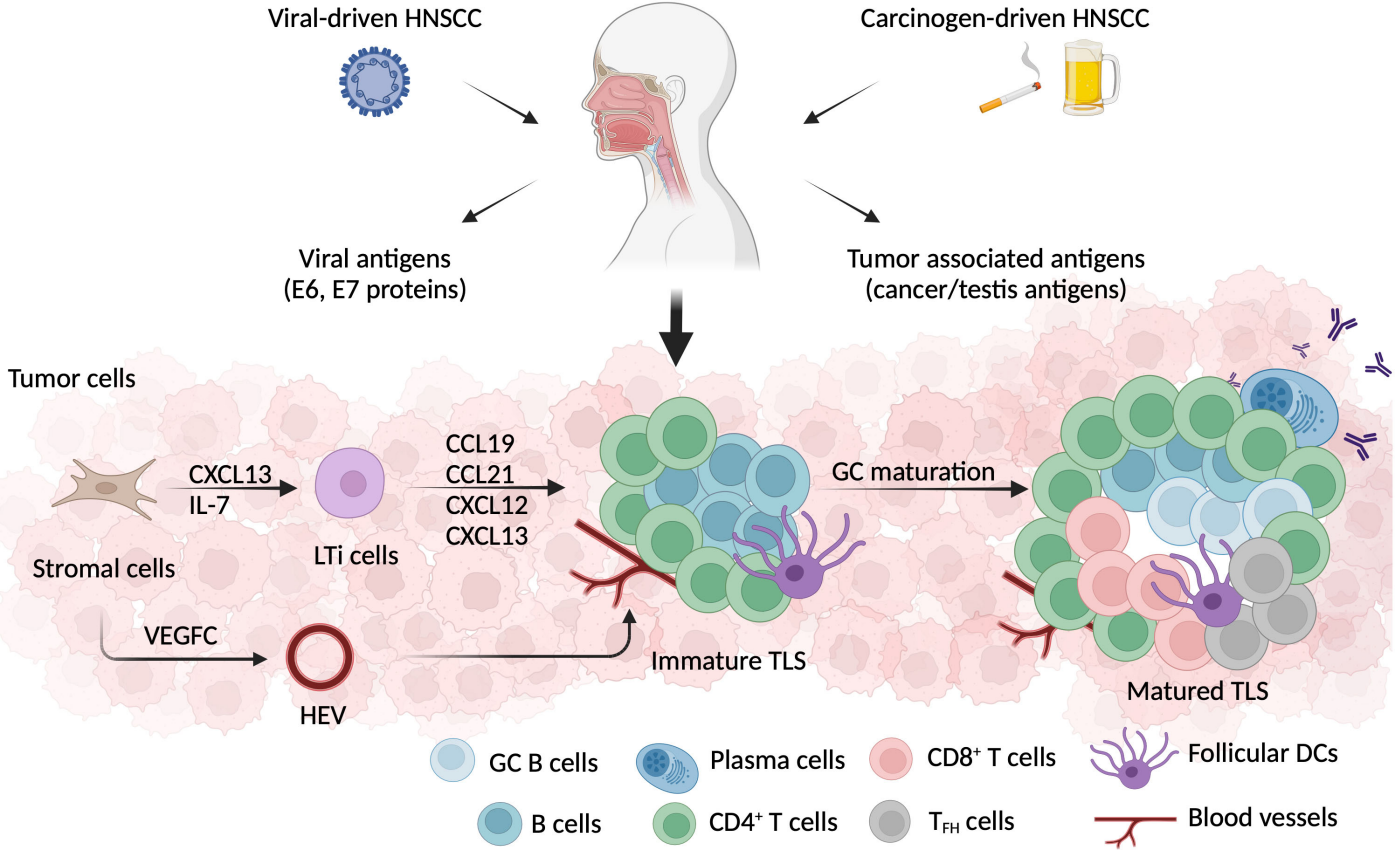
TLS formation in HNSCC. HNSCC can be induced by either viral infection, or environmental carcinogens, such as smoking or excessive alcohol consumption. Besides tumor associated antigens, the HPV+ HNSCC microenvironment also exposes E6 and E7 viral antigens. TLS, a crucial ectopic lymphoid cell formation, develops in the early stage of tumorigenesis. Local stromal cells that have been activated secret CXCL13 and attract lymphoid tissue inducer (LTi) cells. LTi cells produce a series of chemokines, including CCL19, CCL21, CXCL12, and CXCL13, which aid in the recruitment of LTi cells and lymphocytes. Additionally, stromal cells secrete VEGF-C, which promotes HEV development in the vicinity of TLSs. After germinal center (GC) maturation, a well-defined TLSs is formed with a CD3+ T cell zone composed of CD4+, CD8+, and TFH cells, and a CD20+ B cell zone comprising GC B cells, memory B cells, and plasma cells. Besides lymphocytes, follicular DCs, fibroblasts, and neovascular cells are also involved in the process of TLS formation.

The recruitment of lymphoid tissue inducer (LTi) cells to the inflamed area initiates TLS neogenesis. CD4^+^ T cells, CD21^+^ follicular DCs, and stromal cells are capable of producing CXC-chemokine ligand (CXCL) 13 and IL-7, which aid in this process (107, 108) (**Figure 3**). Main LTi cells include IL-21 producing Th17 cells (109, 110), TIL-B cells (111), and M1-polarized TAMs (112). In virus infection, CD4^+^ Th cells partially polarized into IFN^+^IL-1^+^ T_FH_ cells, which stimulate CXCL13 production and support ectopic GC formation (113, 114). Whereas in both breast cancer and nasopharyngeal carcinoma, CXCL13-producing CD4^+^ T cells with either PD-1^hi^T-bet^hi^BCL6^low^CXCR5^-^ or PD-1^hi^CXCR5^-^ phenotype prime TLS neogenesis (115, 116).

After being recruited into the local region, LTi cells communicate with stromal cells via lymphotoxin (LT) α1β2 –LTβR interaction (117), allowing them to produce vascular endothelial growth factor (VEGF) C, to induce high endothelial venule (HEV) formation (118) (**Figure 3**). Then, HEVs serve as entrances for TILs to enter the TLS (119). LTi cells additionally secrete IL-17, which induces the production of CXCL12, CXCL13, CC-chemokine ligand (CCL) 19, and CCL21. This chemokine cocktail stimulates LTα1β2 expression on TILs, and reprograms them into mantle T or B cells (120, 121). The complete TLS neogenesis process is analogous to the formation of human SLOs. An entirely developed TLS, referred to as a secondary follicle-like TLS, encompasses an active GC zone populated by CD23^+^ B cells. On the contrary, an immature TLS (referred to as primary follicle-like TLS) consists of B cell clusters interlinked by FDC networks but lacks GCs (122).

A 12-chemokine gene expression signature (GES) associated with TLS development was identified in multiple solid tumors (103, 123–125). Among these, CCL19, CCL21, and CXCL13 stand out as crucial chemokines for TLS neogenesis. Later, a pan-cancer assessment of gene signatures from The Cancer Genome Atlas (TCGA) database indicated that this 12-chemokine GES is substantially related with TLS-associated cell types, including B lineage cells, T cells, and myeloid cells (103). The differential expression of the 12-chemokine GES in pan cancers demonstrates that TLS abundance is greatly reliant on cancer heterogeneity. Tumors situated in immunologically privileged sites (brain or eyes), or with severe fibrotic stroma (pancreatic cancer), typically have a low expression of the TLS signature, whereas HNSCC has a moderate to high expression (126). This result is consistent with several clinical studies (127–129). In a study of oral squamous cell carcinoma (OSCC) patients, 90.7% (68 of 75) of all cancer samples contained HEV invasion (128). CXCL12, CCL19, and CCL21 were significantly overexpressed in HEV^+^ cases, as were CD3^+^ T cells, CTLs, and CD20^+^ B cells (129). In another OSCC cohort, 26.8% (45 of 168) TLS^+^ patients were identified using immunohistochemical (IHC) staining (127).

TLSs can be detected in the stroma, invasive margin, and tumor core (130, 131). For HNSCC patients, TLSs are more prevalent in the invasive margin (23, 132). In a study of OSCC patients, intratumoral TLSs were observed in 33.8% (22 of 65) of tumor specimens, while peritumoral TLSs were observed in 75.4% (49 of 65) of tumor specimens (132).

### 3.2 Main effector B cells within TLSs

#### 3.2.1 GC B cells

Increased numbers of TILs and TIL-B cells have long been noticed in the TME of HPV^+^ as compared to HPV^-^ HNSCC (23, 133). However, instead of uncovering a T-cell-driven TIL signature that separates HPV^+^ from HPV^-^ HNSCC, adjusting the number of TILs revealed a distinct set of B-cell associated genes which are highly expressed in HPV^+^ HNSCC, including *BCL2, ADAM28, CD200, ICOSLG and SPIB* (133). Among those, *ICOSLG* and *SPIB* are linked to activated B-cells within GCs (134, 135).

GC is the foundation of a matured lymphoid structure (136). It is the domain for B cells to undergo clonal expansion and receptor diversification, producing affinity-matured antibody-secreting plasma cells that effectively recognize the cognate antigens or memory B cells which sustain a durable humoral immunity. In SLO neogenesis, a GC is composed of a dark zone, where B cells undergo clonal expansion and somatic hypermutation (SHM), and a light zone, where B cells further interact with T_FH_ cells for activation and affinity maturation (137). It was recently found that tumor-infiltrating GC B cells in HPV^+^ HNSCC have distinct waves of gene expression consistent with the dark zone, the light zone, and the transitional state, similar to SLOs (2). Moreover, comparing TLS^+^ and TLS^-^ tumor tissues elucidated a B cell-related gene signature ranging from naïve B cells to terminally differentiated plasma cells, which further supports the concept of *in situ* B cell maturation within the TLSs (138).

GC B cells also express several important genes. For example, *SEMA4A*, a membrane-bound glycoprotein for T cell co-stimulation, was found in GC B cells of HNSCC patients (2, 139). The interactive ability of SMEA4a with endothelial and T cells facilitates TLS generation from unmatured immune aggregates (140, 141). Besides, AID, a critical enzyme for SHM and class-switch recombination of immunoglobulin genes, and BCL-6, the transcription factor involved in the late stage of B cell maturation, are also detectable in GC B cells and may be indicators of GC maturation (2, 136, 142).

The cellular compositions of a matured TLS are a core of lymphatic cells, follicular DCs, and fibroblasts, with surrounded neo-vessels for nutrition supply, and non-hematopoietic stromal cells to support the structure (143). T cells with effector memory phenotypes, CD4^+^ T_FH_ cells, B cells with antigen-presenting function, antibody-producing plasma cells, and memory B cells are all generated within TLSs, and play a significant role in anti-tumor immunity (**Figure 3**).

#### 3.2.2 Plasma cells

Plasma cells are one of the most important anti-tumor effector B cells, due to their ability to release antibodies against TAAs. Tumor-infiltrating plasma cells either aggregate in the interspace of TLSs, or disperse in the tumor stroma, forming cellular clusters (116, 136, 144, 145). Recent research by Meylan et al. demonstrates that, following terminal maturation within TLSs, IgG^+^ and IgA^+^ plasma cells could be distributed into the tumor milieu by a network of CXCL12^+^ fibroblasts (138).

The presence of plasma cells both positively correlates and reinforces the cytotoxic effect of CTLs (145). CXCL13-producing PD-1^hi^CD8^+^ T cells and bystander CD8^+^ T cells in close proximity to the CD20^+^ B cell zones of TLSs can detect a broad spectrum of cancer-irrelevant epitopes (146, 147). HPV, Epstein-Barr virus (EBV), human cytomegalovirus, and influenza virus are among the epitopes identified by bystander CD8^+^ T cells. Increased cytolytic activity in tumor patients with early virus infection suggests that bystander CD8^+^ T cells may be produced shortly after the B cell-dominated humoral immune response against viral antigens (148). In nearly all HPV^+^ HNSCC patients, antibodies against viral antigens were detected by serological analysis employing patient serum (149). For patients with high-grade cervical intraepithelial neoplasia, therapeutic vaccination against the E6 and E7 proteins of HPV-16 and HPV-18 promoted TLS neogenesis beneath the neoplasm (150). Clonally expanded T cells were discovered within lesions, which were possibly educated in TLSs. When TIL-B cells or plasma cells co-existed with T cells, the prognostic relevance was substantially higher, according to a meta-analysis of several forms of human cancer (151).

In contrast, it appears that the humoral immune responses in HPV^-^ HNSCC are more diverse (152, 153). Lechner et al. discovered that antibody responses to TAAs were more frequently observed in HNSCC patients with advanced stages (UICC stage III/IV) or the HPV^-^ status (154). Interestingly, only TAA-specific IgG1 antibodies produced by plasma cells can engage in complement activation, ADCC and ADCP (138, 155, 156), and improve the antigen presentation ability of DCs (157).

Non-tumor-specific, antigen-free IgG3 antibodies bind with high affinity to Fcγ receptors, thereby occupying the binding domains of TAMs and NK cells, consequently hindering the interaction with tumor cells as well as ADCC and antibody-mediated phagocytosis (158). In contrast, antigen-free IgG1 antibodies do not occupy the Fcγ receptors on TAMs and NK cells, allowing them to attach to tumor-associated IgG1 or IgG3. The allosteric increase of antigen-bound IgG1 also improve ADCC and phagocytosis (159, 160). Notably, in a pan-cancer study of TCGA database, the IgG3-1 switch is favorably associated with prognosis in patients with a high SHM rate, highlighting the significance of SHM in tumor immunology (161).

IgA is another antibody isotype within the TME, despite the non-mucosal nature of most cancers (65, 138). A high IgA level is related with an immunosuppressive milieu (65, 73, 103, 162). After encountering TGF-β-secreting Treg cells, plasma cells undergo class switch and produce IgA (103, 162). The IgA-producing plasma cells can also co-express IL-10 and PD-L1, which suppress CTL responses, induce effector T cell exhaustion, and accelerate tumor development (65, 73). Intriguingly, a protective humoral response of polyclonal IgA was revealed in ovarian cancer, since it binds to tumor-expressed polymeric IgA receptors (163).

Also identified intratumorally were IgM, IgE, and IgD deposits. IgM and IgD immunoglobulins are poorly expressed in the TME (164), and they are frequently associated with Breg cells (61, 63, 74). A high proportion of IgE or IgD is related with a poor prognosis in melanoma, but not in patients with other types of cancer (63, 136, 165).

#### 3.2.3 Memory B cells

Memory B cells are capable of memorizing antigens and sustaining a lasting immune response. Generated from naive B cells, they constitute the majority of TIL-B cells. In the TME, atypical memory B cells with either antigen-presenting or direct tumor-killing phenotypes have been identified (12–16, 142, 151, 166, 167).

B cells could demonstrate adaptive antigen-presenting phenotypes, functioning effectively as antigen-presenting cells (APCs). Their interactions with T cells are particularly noteworthy, as they proficiently convey antigens and facilitate optimal T cell function (12–16, 122, 167). The competence of B cells as professional APCs is underpinned by their expression of peptide-loaded MHC molecules, costimulatory signals, and cytokine secretion (168). B cells can process and present antigenic peptides via both MHC class II to CD4^+^ T cells (8), and cross-presentation of peptide-MHC I complexes to CD8^+^ T cells (10, 11).

Exploring physiological contexts, marginal zone B cells characterized by B220^+^CD93^+^CD21^+^CD23^-^IgM^+^ expression have been observed to acquire dendritic cell functions through trogocytosis (169). This allows them to exhibit pMHC II-C3dg complexes on their cell membranes, thereby facilitating effective antigen presentation to T cells. Similarly, investigations into rheumatoid arthritis have revealed a CD21^low^CD86^+^ memory-like (IgD^-^CD27^+^) B cell subset with elevated expression of MHC class I and II, suggestive of potent APC capabilities (170). Interestingly, this same B cell phenotype has been identified in TLSs across diverse tumor types, including HNSCC, emphasizing their potential role in antigen presentation to T cells within cancer (167). Additional studies of TIL-B cells have consistently demonstrated elevated levels of MHC class I and II expression, as well as the presence of key costimulatory molecules like CD40, CD80, CD86, and ICOSL, further supporting their enrichment within TLSs (12–16).

The functional prowess of TIL-B cells in inciting T cell effector responses is exemplified in ovarian and liver cancer, where atypical CD20^+^CD27^-^IgG^+^ memory B cells colocalizing with CD8^+^ T cells had the ability to present antigens, and the coexistence of both cells benefited prognosis (142, 151). Patients with lung cancer who had an abundance of intra-TLS B cells had an elevated CD4^+^ TCR clonality and a heightened T cell-dependent B cell response (171). Also in lung cancer, isolated TIL-B cells were found to induce CD4^+^ T cell expansion in response to tumor lysate or cancer-testis antigen (172). Intriguingly, CD4^+^ T cells exposed to activated (CD69^+^HLA^-^DR^+^CD27^+^CD21^+^) versus exhausted (CD69^+^HLA-DR^+^CD27^-^CD21^-^) TIL-B cells displayed a skew towards Th1 versus Treg phenotype. Another study with the HPV^+^ OPSCC demonstrated that, the interactions between CD20^+^ TIL-B cells and CD8^+^ T cells positively correlated with the abundance of HPV-specific CD8^+^ clones, suggesting potential roles of TIL-B cells in supporting CD8^+^ T cell responses (101).

Notably, naïve T cells are mainly situated within lymphoid aggregates like TLSs, compared to the tumor stromal. The study by de Chaisemartin et al. quantified that, TLS T cell zones contained 66% memory cells and 34% naïve T cells, whereas naïve T cells were rarely seen in the other areas of tumor . Engelhard et al. summarizes the necessary prerequisites for the recruitment of naïve T cells in the tumor bed (173, 174). Naïve T cells were previously not considered as present in the TME, as they express L-selectin and CCR7, which are attracted to lymphoid structures but not peripheral tissues. The successfully recruitment of naïve T cells in engineered or unmanipulated tumors are highly dependent on the tumor-associated blood vessels that express PNAd and CCL21 (175–177). The latter are expressed specifically on HEVs, which is an important vascular structure that support TLS formation. Moreover, compared to TLS^low^ tumors, TLS^hi^ cancers overexpress genes involved in T cell activation, chemotaxis, cytotoxicity, and Th1 cell skewing (119, 178–180), which further supports the hypothesis that TLSs are the initial site for T and B cells interaction and maturation.

In addition to the ability to deliver antigens, memory B cells with direct tumor-killing capabilities have been found in TLSs. Memory B cells in hepatocellular carcinoma (HCC) expressed large quantities of tumor-killing cytokines, including IFN-γ, IL-12p40, GrB, and TRAIL (166). The characteristics and roles of the predominant TIL-B cell groups observed in the tumor milieu are summarized in **Table 3**.

**Table 3:** Phenotypes and functions of important TIL-B cells.

TIL-B cells	Phenotype	Functions
GC B cell	CD19^+^CD20^+^CD27^+^CD38^+^CD10^+^IgD^-^; BCL6, AID, SEMA4A, and Ki67 expression	Dark zone GC B cells undergo clonal expansion and somatic hypermutation before migrating into the light zone and interacting further with T_FH_ cells and follicular DCs for affinity maturation. The delivery of transcription factors by mature follicular DCs and T_FH_ cells determines whether GC B cells become memory B cells or terminal differentiated plasma cells.
Memory B cell	CD19^+^CD20^+^CD24^+^CD27^+^CD38^low^ (atypical CD27^-^ phenotype)	Sustain long-term immune response.
antigen-presenting phenotype	MHC-mediated; CD40/CD80/CD86 expression	Present antigens to T cells, and stimulate the activation and effector functions of cytotoxic CD8^+^ T cell.
direct tumor killing phenotype	IFN-γ, IL-12p40, GrB and TRAIL expression	Direct tumor cell killing effect.
Plasma cell	CD19^low^CD20^low^CD24^-^CD27^hi^CD38^hi^, IgG or IgA expression	Produce antibodies which recognize the tumor-associated antigens, cause antibody-dependent cellular cytotoxic and phagocytosis, activate the complement system, and augment antigen presentation by DCs.
Regulatory B cell	Multiple phenotypes	Produce immunosuppressive cytokines, such as IL-10, IL-35, and TGF-β; induce effector T and B cell malfunction; promote the proliferation of Treg cells and MDSCs; promote tumor progression and metastasis.

TIL-B cells, tumor-infiltrating B cells; GC B cell, germinal center B cell; DC, dendritic cell, T_FH_ cell, follicular helper T cell; MHC, major histocompatibility complex; AID, activation-induced deaminase; GrB, granzyme B; Treg cell, regulatory T cell; MDSC, myeloid-derived suppressor cell; hi, high; dim, medium.

### 3.3 The relation of TLSs with clinicopathological features

The presence of TLS is associated with a higher tumor grade, clinical stage, and TILs infiltration (181–185). However, the percentage of HEV is higher in T1/T2 stage HNSCC patients (128, 129). At metastatic sites, the density of TLSs remains positively related to the number of TILs within tumor beds. The characteristics of effector T and B cells in metastatic cancers closely resemble those in primary tumors, encompassing traits such as effector T cell infiltration, CTL skewing, effector B cell clonal expansion, rearrangement of immunoglobulin genes, SHM, and isotype switching (164, 186–191).

A favorable impact of HEV/TLS density on survival was observed in several studies of HNSCC patients (127, 129, 132, 133, 192). HNSCC patients with enriched intratumoral HEV/TLS in the primary site had better overall survival and disease-free survival (129, 132). In patients with early-stage HNSCC, CD20^+^ TIL is a good prognostic factor (193). When analyzing the metastatic LNs of HNSCC patients, a high frequency of TIL-B cells was also associated with an improved disease-free survival (194).

However, few discordant results appeared in studies of other cancer entities. In HCC, TLSs located in the tumor-adjacent inflamed area were related with tumor progression (195, 196). In HER2^-^ breast tumors, TLSs were related with lymphatic invasion, higher pathological nodal stage, and nodal involvement (182). Given that TLSs are locations where immune responses are initiated, yet not fully realized, they might not indicate the most reliable prognostic or predictive values. Alternatively, lympho-myeloid aggregates, such as plasma cell zones, have recently been proposed, calling for precise definitions and objective measurements in the near future (197). Meanwhile, in the research conducted by Noel et al, an active TLS associated with favorable prognosis is characterized by an increased proportion of functional Th1-oriented PD-1^hi^ICOS^int^ T_FH_ TIL and a higher effector versus regulatory TIL ratio (198). Meanwhile, the CD25^+^CXCR5^+^GARP^+^FoxP3^+^ follicular regulatory T (T_FR_) cells prohibits the T_FH_ TIL from faciliting the antibody production capacity of plasma cells, and a favorable functional T_FH_ over T_FR_ ratio would generate a Th1 microenvironment that governs active TLS maturation. Given the potential for certain TLSs to be halted in their developmental process due to immunosuppressive elements within the TME (199), and the likelihood of others regressing once their initiating antigens are eliminated (200), there is a pressing need to delve into a more all-encompassing understanding of the stimuli and mechanisms governing TLS formation and maturation.

In conclusion, TIL-B cells within TLSs are expected to be shielded from environmental challenges due to their unique spatial configuration. They interact directly with effector TILs, allowing them to mature into immunocompetent cells with anti-tumor effects. In an unmatured TLS, GC B cells first appear. Following clonal expansion and receptor diversification, memory B cells with different functional capacities, and matured plasma cells are present. These effector B cells efficiently produce antibodies, recognize TAAs, communicate with effector T cells, and maintain the anti-tumor immune response. Even if the link between TLSs and clinicopathologic characteristics of patients is still debatable, one cannot overlook the beneficial mechanisms of TLSs. Future analyses may also consider the location and proximity of TLSs, GC maturation, compositional zone liveness, and antibody isotypes produced by local plasma cells.

## 4 Targeting TIL-B cells for immunotherapy

Immunotherapies for cancer are currently predominated based on ICB treatment, which focuses mainly on reserving CTL effector functions. However, only a small subset of HNSCC patients may benefit from this approach, with only 20% of patients exhibiting an initial response to the PD-1 therapy (201–203). Moreover, some patients eventually develop acquired resistance to this therapy.

Given the limitations of single-agent ICB therapy, exploring TIL-B cells as possible targets for novel immunotherapy paradigms could be a valuable complement. The cellular and humoral immune resistance of cancer always work in tandem. Remarkably, individuals diagnosed with HNSCC who demonstrate either previous HPV infection (resulting in viral antigens) or a high tumor mutation burden (leading to increased TAAs) have shown enhanced responsiveness to ICB therapies (204, 205). Additionally, it has been observed that PD-L1, the ligand commonly expressed on APCs and tumor cells, is expressed in certain Breg cells, further highlighting the potential role of TIL-B cells in modulating the immune response in cancer.

Despite the limited availability of B-cell-specific treatments for solid cancers, numerous immunotherapeutic agents have been shown to affect TIL-B cells and TLSs formation. In this section, we discuss the immunotherapeutic approaches surrounding TIL-B cells that either promote TLS neogenesis and functions or reduce the immunosuppressive potential of Breg cells. The immunotherapies mentioned in this section are summarized in **Table 4**.

**Table 4:** Targeting TIL-B cells for immunotherapy.

Agent	Phase	Description	Related functions
GVAX	Neoadjuvant clinical trial (NCT02451982 and NCT02648282)	Therapeutic cancer vaccine	Induce GM-CSF secretion, promote TLS formation, facilitate TIL infiltration, foster effector T cell activation and cellular communications (206, 207).
Anti-VEGFR	Preclinical	Angiogenesis modulator	Used as an adjuvant to enhance ICB therapy, induce HEV formation, improve anti-PD-L1 treatment, promote TIL infiltration (208).
GSK3359609	Phase II/III clinical trial (NCT04128696 and NCT04428333)	ICOS agonist	Promote memory and effector T cell development, induce specific humoral immune responses (209).
Engineered DCs	Preclinical Phase I clinical trial (NCT01574222)	Targeted cytokine/chemokine delivery system	Targeted expression of ideal cytokines or chemokines, initiate TLS resemble, enhance Th1 cell skewing, promote CD8^+^ CTL infiltration (210–212).
CXCL13-coupled CpG-ODN	Preclinical	Therapeutic cancer vaccine	Targeted delivering of stimulatory CpG-ODN to effector B cells, activate effector TIL-B cells while block the generation of Breg cells, promote GrB-expressing CTLs (78).
Nanoengineered synthetic immune niches	Preclinical	Synthetic scaffolds with modified TILs and TAA-pulsed DCs	Artificial-designed “TLSs” for reprogramming immunosuppressive TME (213–215).
Tirabrutinib	Preclinical	BTK inhibitor	Suppress Breg cell accumulation, reduce IL-10 and IL-35 secretion, foster CD8^+^ CTL accumulation (68).
Cobimetinib	Preclinical	MEK inhibitor	Reduce Breg cell infiltration, interrupt chronic BCR signaling, spare anti-tumor humoral immunity (216).
Resveratrol	Preclinical	STAT3 inhibitor	Hamper Breg cell generation, downregulate TGF-β secretion, impair the conversion of FoxP3^+^ Treg cells (75, 217)
Lipoxin A4	Preclinical	Lipid mediator	Suppress Breg cell induction, reduce Treg cell proliferation, relieve CTL activities (218).
IL-35 neutralizing antibody	Preclinical	IL-35 antidote	Reduce PD-L1^+^ Breg cells, increase CD8^+^CXCR3^+^CCR5^+^ T cells, improve anti-PD-1 treatment, enhance IgG- and IgA- expressing plasma cell differentiation (53, 219, 220).

TIL-B cell, tumor-infiltrating B cell; GM-CSF: granulocyte-macrophage colony-stimulating factor; VEGFR, vascular endothelial growth factor receptor; ICB, immune checkpoint blockage; TAA, tumor-associated antigen; ICOS, inducible T cell costimulator; DC, dendritic cell; TLS, tertiary lymphoid structure; IL-10, interleukin-10; GrB, granzyme B; MEK, mitogen/extracellular signal-regulated kinase; BCR, B cell receptor; BTK, Bruton’s tyrosine kinase; ODN, oligonucleotide.

### 4.1 Foster TLS formation

High amounts of TIL-B cells and TLSs are associated with better responses to ICB therapies (14–16, 148, 221, 222). In HPV^+^ HNSCC, patients who reacted to radiotherapy in conjunction with PD-1 antagonist showed increased GC formation, effector B cell generation, and enhanced IgG and IgM antibody responses (221). For non-small cell lung cancer patients, who responded to a neoadjuvant PD-1 antagonist, significant enrichment of TLSs was found in their tumor microdissections (223). The presence of pre-treatment PD-1^hi^CD8^+^ T cells within TLSs was also a predictor of anti-PD-1 response (147).

Several therapeutic cancer vaccines have demonstrated their efficacy. GVAX is a cancer vaccine that was genetically modified to induce the granulocyte-macrophage colony-stimulating factor (GM-CSF). Extensive TLS formation in patients with pancreatic cancer was found after GVAX treatment, and was associated with a good prognosis (NCT00727441) (206, 207). Subsequent gene expression analysis identified pathways regulating immune cell activation and communication. Treg cell suppression, Th17 cell activation, and elevated effector T cells to Tregs ratio were also noticed. Additionally, unmethylated cytosine-phosphate-guanine oligodeoxynucleotide (CpG ODN) is a type of tumor nanovaccines which utilized CpG nanoparticles as adjuvants (224). The water-soluable CpG can enter B cells and plasmacytoid DCs, triggering strong innate and adaptive immune response. When a CXCL13-coupled CpG ODN was applied in mice with 4T1 breast cancer metastasis, it successfully stimulated effector TIL-B cells via the CXCL13-CXC5R interaction, promoted GrB-expressing CTLs, without stimulating the CD20^low^ Breg cells (78).

Besides, several combination therapies of ICBs and targeted agents have received satisfied results. ICBs and vascular targeting peptide combination therapy increased both the *de novo* formation of TLSs, and the effector T cell activation (225). A combined therapy of anti-VEGFR2 and anti-PD-L1, induced HEV formation, promoted cytolysis, and transformed the immunosuppressive tumor into an immune-active phenotype (208). Those findings point out that the pharmacological approaches to foster TLSs might serve as good supplements for ICB therapies, as they are likely to subvert the immune-resistant tumors to immunogenic types.

Studies have investigated the possible strategies to combine T cell-reinvigorated immunotherapies with a great harvest of TLSs. The inducible T cell costimulator (ICOS) is a co-stimulatory factor expressed on activated T cells, which plays an important role in cell-cell signaling, CTL formation, and Treg cell activation (209). Its ligand ICOSL is highly expressed in HPV^+^ HNSCC and is associated with activated TIL-B cells within GC (133). The effect of ICOS-agonist GSK3359609 in combination with anti-PD-1 pembrolizumab has been tested on HNSCC patients in the INDUCE-1 study. Anti-PD-1/PD-L1-naïve HNSCC patients who received the combined therapy (n = 34) had a significantly higher overall response rate and disease control rate compared to patients received GSK3359609 alone (n = 17) (226). Now the effect of GSK3359609 has been continuously studied in two phase II/III clinical trials (INDUCE-3 trial, NCT04128696 and INDUCE-4 trial, NCT04428333), comparing GSK3359609 plus pembrolizumab vs placebo plus pembrolizumab with and without the combination of 5-fluorouracil (5-FU)-platinum chemotherapy.

Additionally, the exploration of cell-based cytokine/chemokine delivery systems has provided valuable insights. Since endothelial cells express IL-36γ to sustain follicular B cell functions (227), engineered DCs were programmed to express both IL-36γ and the T cell-specific T box transcription factor (T-bet) and then delivered into a sarcoma mouse model (210, 211). This IL-36γ-dependent T-bet therapy enhanced both Th1 cell skewing and TLS neogenesis. Another compelling evidence comes from a phase I clinical trial (NCT01574222), where patients with advanced lung cancer received injections of CCL21-expressing engineered DCs. Evidently, this intervention triggered systemic anti-tumor responses, leading to heightened infiltration of CD8^+^ TILs (212).

Emerging technologies such as synthetic scaffolds have been developed with the purpose of cultivating modified LNs-derived cell lines along with tumor antigen-pulsed DCs in biocompatible scaffold materials (213, 214). This nanoengineered synthetic immune niches have the potential to function as a versatile platform for immune-reprogramming (215). Introducing a stromal cell line derived from LN-induced TLSs has demonstrated the enhancement of the anti-tumor immune response, leading to increased TILs in a mouse model of colon cancer (228).The previously mentioned 12-chemokine GES can be harnessed for the construction of ectopic designer TLSs (103, 123–125, 229).

The treatment methods related to the development of TLSs still have broad prospects. For example, methods could be explored to facilitate GC maturation, foster the entry of matured plasma cells into tumor tissue, and the secretion of tumor-associated antigens. Since SEMA4A is a marker for both early-stage and functional TLSs, promoting SEMA4A expression on TIL-B cells might serve as a potential therapeutic option (2, 140, 141). On the other hand, diet, CD20 antagonist rituximab, chemotherapy, and corticosteroids can eradicate the development of GCs, decrease TLSs density, and might impair the positive therapeutic impact (126, 230, 231).

### 4.2 Hamper Breg cells

Breg cells have been used as novel targets in cancer treatment because of their immunosuppressive and tumor-promoting activities. The presence of PD-L1 on some Breg cells also confirms their participation in ICB treatments (62, 63).

B-cell depletion therapies lacking more precise targeting, such as the CD20 antagonist rituximab, are scarcely applicable in solid cancers. It can enhance cancer progression and metastasis by evoking CD20^low^ Breg cells (78, 232). Conversely, a more promising approach for Breg depletion therapy involves exploring alternative Breg-specific markers. For example, CD200 is a type I membrane-associated glycoprotein related to an immunoregulatory signaling pathway, which is detectable across multiple haemalogic malignancies and solid cancers (233). In HPV^+^ HNSCC patients, CD200^+^ expressing Breg cells were identified (133). However, Samalizumab, the anti-CD200 monoclonal antibody, was tested in haemalogic malignancies including B-cell chronic lymphocytic leukemia and multiple myeloma (NCT00648739), and solid cancers (NCT02987504) without satisfied outcome. Multiple adverse outcomes were reported in patients, including skin rashes, joint stiffness/pain, headaches, and blood disorders (234). Since CD200 is also widely expressed in normal cells of both haematopoietic and non-haematopoietic origin, one could speculate the potential toxicities (235). Therefore, opting for Breg cell depletion therapy must be used with caution.

In lieu of depleting Breg cells, inhibitors of MEK, BTK, and STAT3 have been reported to hinder Breg formation and promote anti-tumor immunity across various mouse tumor models. Tirabrutinib, a small molecule BTK inhibitor, effectively inhibits aberrant BCR signaling in B cell-related cancers. In PanIN-bearing mice, tirabrutinib was found to suppress CD5^+^CD1d^hi^ Breg cells accumulation, reduce IL-10 and IL-35 secretion, increase CD8^+^IFN-γ^+^ CTLs, and attenuate PanIN growth (68). Mitogen-activated protein kinase (MAPK) kinase (MEK) inhibitor is a targeted therapeutic agent for tumors with *BRAF* or *KRAS* oncogene mutations (236, 237). Cobimetinib, a MEK inhibitor, decreases the number of T2-MZP Bregs, B10 Bregs, and TIM1^+^ Bregs in tumor-draining LNs of mice with colorectal cancer by interrupting chronic BCR signaling, while sparing the anti-tumor humoral immunity of functional B cells (216). While in 4T1 lung-metastatic breast adenocarcinoma, resveratrol, a phytoalexin and antioxidants, hampers Breg cell generation, downregulates TGF-β secretion by inactivating STAT3, and concurrently impairs the Breg cell-induced conversion of FoxP3^+^ Treg cells (75, 217). Nevertheless, cautious administration is required to mitigate potential non-targeted and wide-spread dysfunction of TIL-B cells.

Potential therapies for the prevention or conversion of Breg phenotypes are also available. In multiple murine cancer models, Lipoxin A4, a metabolite of arachidonic acid with anti-inflammatory characteristics, was found to selectively suppresses B10 Breg induction (218). This is accompanied with reduced Treg cells in the tumor tissues and draining LNs, while the proliferation, differentiation, and GC formation roles of effector B cells are reserved. Moreover, in mice with pancreatic cancer, neutralizing IL-35 reduces the frequency of PD-L1^+^ Breg cells, stimulates CD8^+^CXCR3^+^CCR5^+^ T cell production, and overcomes resistance to anti-PD-1 immunotherapy (53, 219). Also in pancreatic cancer, the B cell-specific deletion of IL-35 has been linked to enhanced plasma cell differentiation and the production of anti-tumor IgG and IgM antibodies (220).

Taken together, these data suggest that promoting TIL infiltration, activation, and differentiation, or promoting TLS neogenesis and function or manipulating Breg cells to inhibit cancer progression holds promise for immunotherapy of solid tumors, including HNSCC. On top of that, more studies are urgently needed for a comprehensive understanding of the factors that drive the assembly of TLSs, and the phenotypical and functional differences of Breg cells, in order to develop specifically-designed TIL-B cells targeting immunotherapeutic approaches.

## 5 Discussion

Largely overlooked in the past but now increasingly in the focus of recent research, TIL-B cells are gaining traction as key cellular players in the TME that can elicit either pro- or anti-tumor effects.

Scattered TIL-B cells in the TME are more likely to obtain a regulatory effect that inhibits the activation and function of effector T cells, hence dominating an immunosuppressive role. However, when TIL-B cells are spatially organized in an immune-privileged site, also known as TLSs, they develop anti-tumor capabilities due to their exceptional intercellular contacts with T cells and other effector TILs. TLS-matured effector B cells can either produce tumor-specific antibodies, thereby aiding in tumor recognition, ADCC, ADCP, and activation of the complement cascade, or deliver TAAs or viral antigens to CTLs and maintain long-term immunological memory.

As novel technologies continue to emerge, such as single-cell RNA-sequencing, spatial transcriptomics, and multiplex imaging, a greater understanding of the diverse subtypes of TIL-B cells, including their BCR clonality, spatial distribution, and cellular interactions, will be established. By expanding our knowledge of the fascinating role of TIL-B cells in the HNSCC tumor microenvironment, tailored therapeutic strategies can be designed for personalized clinical applications. Ultimately, the potential for manipulating TIL-B cells to enhance anti-tumor immune responses provides exciting opportunities for the future of cancer immunotherapy.

## Author contributions

JB wrote the manuscript. CB, JH and AB reviewed and edited the manuscript. All authors contributed to the article and approved the submitted version.

## Glossary

**Table:**

HNSCC	head and neck squamous cell carcinoma
TSCC	tongue squamous cell carcinoma
OSCC	oral squamous cell carcinoma
HPV	human papillomavirus
EBV	Epstein-Barr virus
TME	tumor microenvironment
Breg cell	regulatory B cell
Treg cell	regulatory T cell
CTL	cytotoxic T lymphocyte
SHM	somatic hypermutation
SLO	secondary lymphoid organ
TLS	tertiary lymphoid structure
TIL	tumor-infiltrating lymphocyte
PD-1	programmed cell death protein 1
PD-L1	programmed cell death 1 ligand 1
CTLA-4	cytotoxic T-lymphocyte associated protein 4
GC	germinal center
ADCC	antibody-dependent cellular cytotoxicity
ADCP	antibody-dependent cellular phagocytosis
MDSC	myeloid-derived suppressor cell
IL	interleukin
TGF	transforming growth factor
IFN	interferon
IDO	indoleamine 2,3-dioxygenase
Th	helper T cell
DC	dendritic cell
TAM	tumor-associated macrophage
NK	natural killer
APC	antigen-presenting cells
GrB	granzyme B
ADO	adenosine
BTK	Bruton’s tyrosine kinase
TLR	toll-like receptor
T_FH_	follicular helper T cell
T_FR_	follicular regulatory T cell
TCR	T-cell receptor
BCR	B-cell receptor
PanIN	pancreatic intraepithelial neoplasia
T2-MZP	transitional 2-marginal zone precursor
LN	lymph node
LTi	lymphoid tissue inducer
CXCL	CXC-chemokine ligand
TCGA	The Cancer Genome Atlas
GES	gene expression signature
IHC	immunohistochemical
LT	lymphotoxin
VEGF	vascular endothelial growth factor
VEGFR	vascular endothelial growth factor receptor
HEV	high endothelial venule
CCL	CC-chemokine ligand
TAA	tumor-associated antigen
ICB	immune checkpoint blockade
MEK	mitogen/extracellular signal-regulated kinase
T-bet	T cell-specific T box transcription factor
ICOS	inducible T cell co-stimulator
GM-CSF	granulocyte-macrophage colony-stimulating factor
CpG ODN	unmethylated cytosine-phosphate-guanine oligodeoxynucleotide
