# Corrigendum: Exploring the dual role of B cells in solid tumors: implications for head and neck squamous cell carcinoma

**DOI:** 10.3389/fimmu.2023.1327795

**Published:** 2023-11-20

**Authors:** Jiantong Bao, Annika C. Betzler, Jochen Hess, Cornelia Brunner

**Affiliations:** ^1^ Department of Otorhinolaryngology and Head & Neck Surgery, University Medical Center Ulm, Head & Neck Cancer Center of the Comprehensive Cancer Center Ulm, Ulm, Germany; ^2^ School of Medicine, Southeast University, Nanjing, China; ^3^ Department of Otorhinolaryngology, Head and Neck Surgery, Heidelberg University Hospital, Heidelberg, Germany; ^4^ Molecular Mechanisms of Head and Neck Tumors, German Cancer Research Center (DKFZ), Heidelberg, Germany

**Keywords:** head and neck cancer, tumor-infiltrating lymphocytes, regulatory B cells, tertiary lymphoid structures, tumor microenvironment, immunotherapy

In the published article, the reference for 154 was incorrectly written as “Lechner M, Engleitner T, Babushku T, Schmidt-Supprian M, Rad R, Strobl LJ, et al. Notch2-mediated plasticity between marginal zone and follicular B cells. Nat Commun (2021) 12:1111. doi: 10.1038/s41467-021-21359-1”. It should be “Lechner A, Schlößer HA, Thelen M, Wennhold K, Rothschild SI, Gilles R, et al. Tumor-associated B cells and humoral immune response in head and neck squamous cell carcinoma. OncoImmunology (2019) 8:1535293. doi: 10.1080/2162402X.2018.1535293”.

The authors apologize for this error and state that this does not change the scientific conclusions of the article in any way. The original article has been updated.

